# A zero altered Poisson random forest model for genomic-enabled prediction

**DOI:** 10.1093/g3journal/jkaa057

**Published:** 2020-12-21

**Authors:** Osval Antonio Montesinos-López, Abelardo Montesinos-López, Brandon A Mosqueda-Gonzalez, José Cricelio Montesinos-López, José Crossa, Nerida Lozano Ramirez, Pawan Singh, Felícitas Alejandra Valladares-Anguiano

**Affiliations:** 1 Facultad de Telemática, Universidad de Colima, Colima, Colima 28040, México; 2 Departamento de Matemáticas, Centro Universitario de Ciencias Exactas e Ingenierías (CUCEI), Universidad de Guadalajara, 44430 Guadalajara, Jalisco, México; 3 Departamento de Estadística, Centro de Investigación en Matemáticas, Guanajuato, Guanajuato 36023, México; 4 Colegio de Postgraduados, Montecillos, Edo. de México CP 56230, México; 5 International Maize and Wheat Improvement Center (CIMMYT), Km 45, Carretera Mexico-Veracruz, CP 52640, Edo. de México, México; 6 Centro de Investigación y Formación del Pensamiento Libre en México, A.C., Colima, México

**Keywords:** genomic selection, count data, random forest, zero altered Poisson, plant breeding, Genomic Prediction, GenPred, Shared Data Resource

## Abstract

In genomic selection choosing the statistical machine learning model is of paramount importance. In this paper, we present an application of a zero altered random forest model with two versions (ZAP_RF and ZAPC_RF) to deal with excess zeros in count response variables. The proposed model was compared with the conventional random forest (RF) model and with the conventional Generalized Poisson Ridge regression (GPR) using two real datasets, and we found that, in terms of prediction performance, the proposed zero inflated random forest model outperformed the conventional RF and GPR models.

## Introduction

Novel methodologies like genomic selection (GS) proposed by Bernardo (1994) and Meuwissen *et al.* (2001) are gaining popularity in plant breeding because they are revolutionizing the plant breeding paradigm. The basic idea of GS is to perform the process of selection of candidate individuals by only genotyping and phenotyping a reference population and with this information train a statistical model that is then used for predicting genomic breeding values or phenotypic values of a testing (breeding) population that only contains genotypic information. The acceptance and popularity of GS continues to increase since empirical evidence shows that there are no significant differences between the performance of GS and that of phenotypic selection (Roorkiwal *et al.* 2016; Crossa *et al.* 2017; Wolfe *et al*. 2017; Huang *et al.* 2019). Some of the advantages of GS are: (1) it shortens the generation interval, (2) it requires fewer resources, and (3) it reduces the cost per cycle (Farah *et al.* 2016; Crossa *et al.* 2017).

When using GS as a predictive methodology, we need to choose the right model in each circumstance to guarantee an optimal performance. For this reason, nowadays many statistical machine learning models are often used in GS, since there is no universal model that works for all the data at hand (Wolpert and Macready 1997). The development of prediction models for GS is an active area of research that aims to improve the prediction performance of the existing statistical machine learning algorithms in the context of a large number of independent variables (p) and a small sample size (n), of correlated traits and input information from different sources, among others. When the traits are counts, like the number of panicles per plant, number of seeds per plant, number of infected spikelets per plant, days to heading, days to maturity, and days to germination, among others (Montesinos-López *et al.* 2016, 2017, 2020a, 2020b), there are regression models like generalized Poisson regression (Stroup 2012), Bayesian Generalized Poisson regression (Montesinos-López *et al.* 2015, 2016, 2017) and even deep neural networks models (Montesinos-López *et al.* 2020a, 2020b). However, all these models use as a loss function the negative of the log likelihood of a Poisson distribution. Poisson distribution is very popular for count data (that take values of 0, 1, 2,… with an unrestricted upper limit), but has two main disadvantages: (1) it is an intrinsic property of a Poisson distribution that the variance is equal to the mean, and for this reason many times it is unable to capture over-dispersion efficiently, and (2) it cannot efficiently model excess zeros in the response variable.

Another problem of the Poisson family of regression models is that they are parametric models that many times are not efficient for capturing nonlinear patterns. For this reason, many machine learning algorithms have been successfully implemented in GS (Sarkar *et al*. 2015; Stephan *et al*. 2015; Naderi *et al.* 2016; Waldmann 2016; Li *et al.* 2018) to capture nonlinear effects. One of the most popular machine-learning methods is Random Forests (RF, Breiman 2001), which is a tree-based ensemble method for continuous (regression), binary and categorical classification using multiple variables as input (Chen and Ishwaran 2012; Alarcon *et al.* 2015; Li *et al.* 2016). RF has been applied in genome-wide association studies to identify single nucleotide polymorphisms (SNP) associated with phenotypes, and to map QTL on the genome (Brieuc *et al.* 2015; Everson *et al.* 2015; Petralia et al. 2015; Stephan *et al.* 2015; Li *et al.* 2018). In addition, RF has been used for cancer identification and treatment, for epistasis detection (Pashaei *et al.* 2015; Shi and He 2016), for prediction of protein DNA-binding sites from amino acid sequences (Wu *et al*. 2009) and protein-protein interaction sites in sequence (Sikic *et al.* 2009), and for gene network pathway analysis (Pang *et al.* 2006; Wang *et al.* 2010; Chen and Ishwaran 2012).

There is evidence that RF performs better than other methods for binary traits when the sample size is large and the percentage of missing data is low (García-Magariños *et al.* 2009). However, Naderi *et al.* (2016) found that, for binary traits, RF outperformed the GBLUP method only in a scenario combining the highest heritability, the extensive number of markers (50 K SNP chip), and the largest number of QTL. González-Recio and Forni (2011) found that RF performed better than Bayesian regressions in detecting resistant and susceptible animals from based on genetic markers. They also reported that RF produced the most consistent results with very good predictive ability and outperformed other methods in terms of correct classification.

The popular RF models were originally developed for continuous, binary and categorical data. The RF for continuous response variables uses the sum of squared errors (least square) as splitting criteria, while the random forest for binary and categorical data uses the Gini index of the log-likelihood based on a Bernoulli distribution. There are also RF models for count data (Chaudhuri *et al.* 1995; Loh 2002) that can be implanted in R using the package part (Therneau and Atkinson 2019). However, these RF models for count data are not appropriate for counts with excess zeros. For this reason, Lee and Jin (2006) proposed a RF method for counts with an excess of zeros, by building the splitting criterion with the zero-inflated Poisson distribution, but it models both the excess zero part and the Poisson part jointly, which is unlike the basic hurdle and zero-inflated regression models that use two models, thus allowing different covariates’ effects for each part. A common model is based on the assumption that the excess of zeros is generated by an independent random variable. For this reason, conventional regression models for counts with an excess of zeros use a logistic model for predicting excess zeros and a truncated Poisson model for counts larger than zero.

For this reason, in this paper, we present an application of the zero-truncated Poisson random forest with excess zeros proposed by Mathlouthi *et al.* (2019); its building process is similar to the zero altered (or inflated) Poisson regression since two models are used in the building process: one to model excess zeros (zero part) and the other to model counts larger than zero (Poisson part). The proposed method is semi-parametric since it includes only a few assumptions about a specific parametric form. The zero part was modeled using a conventional binary random forest model, while the truncated Poisson part was modeled using an RF with a new splitting criterion based on the zero-truncated Poisson distribution.

## Material and methods

### Univariate ridge regression model

Under this model, the relationship between the response variable that is continuous $\left(y_{i}\right)$ and the input information [$x_{i}^{T}=\left(x_{i1,\;}\ldots ,x_{ip}\right)$ for i=1,…,n] is given by $y_{i}=\beta_{0}+\sum_{j=1}^{p}x_{ij\;}\beta_{j}+e_{i}$, where $e_{i}$ is assumed distributed as normal with mean zero and variance ${(\sigma }^{2})$. The estimates of βs using univariate ridge regression (RR) are obtained by minimizing the following penalized residual sum of squares (loss function):

$$LL=\sum_{i=1}^{n}{\left(y_{i}-\beta_{0}+\sum_{j=1}^{p}x_{ij\;}\beta_{j}\right)}^{2}+\lambda \left(\sum_{j=1}^{p}\beta_{j}^{2}\right)$$

where λ is the tuning hyper-parameter that can be chosen by cross-validation. The optimization of this loss function (LL) was done using the R package glmnent (Lasso and Elastic-Net Regularized Generalized Linear Models) (Friedman *et al.* 2010).

### Univariate generalized Poisson regression model

Since we are in a context where the number of independent variables (p) is larger than the number of observations (n), the penalized loss function for the univariate generalized Poisson regression (GPR) model is equal to:

$$LL=-\sum_{i=1}^{n}\left[-\mu_{i}+y_{i}\;\text{log}\;\left(\mu_{i}\right)\right]+\lambda \left(\sum_{j=1}^{p}\beta_{j}^{2}\right),$$

where LL was derived as the negative penalized log likelihood based on a Poisson distribution, $\mu_{i}=E\left(y_{i}|x_{i}^{T}\right)=\exp(\eta +\sum_{j=1}^{p}x_{ij}\beta_{j})$, represent the inverse link function that is an exponential function and correspond to a log link function, and λ is regularization parameter that can be computed using cross-validation. The type of penalization that contains the loss function is called Ridge penalization since the sum of the squared beta coefficients is taken into account in the penalization term. The loss function was optimized with the R package glmnent and the λ hyper-parameter was estimated with 10-fold cross-validations for both Ridge regression models (RR and GPR). More details about this model can be found in Montesinos-López *et al.* (2020b).

#### Random forests

Random forest (RF) is a modification of bootstrap aggregating that builds a large collection of trees, and then averages out the results. Each tree is built using the least-square splitting criterion (loss function), the usual one when the response variable is continuous. For training data (Breiman 2001), RF takes B bootstrap samples and randomly selects subsets of features as candidate predictors for splitting tree nodes. Each tree minimizes the average loss function in the bootstrapped data and is constructed using the following algorithm:

For b=1,…,B bootstrap samples $\left\{y_{b},X_{b}\right\}$:

Step 1. From the training dataset, draw bootstrap samples of size $N_{\mathrm{train}}$.

Step 2. With the bootstrapped data, grow a random-forest tree $T_{b}$ with the least-square splitting criterion, by recursively repeating the following steps for each terminal node of the tree, until the minimum node size is reached.

1. Randomly draw mtry out of the m independent variables (IVs). mtry is a user-specified parameter.
2. Pick the best independent variable among the mtry IVs.
3. Split the node into two child nodes. The split ends when a stopping criterion is reached, for instance, when a node has less than a predetermined number of observations. No pruning is performed.

Step 3. Output the ensemble of trees ${\left\{T_{b}\right\}}_{1}^{B}$.

The predicted value of testing set (${\hat{y}}_{i}$) individuals with input $x_{i}$ is calculated as ${\hat{y}}_{i}=\frac{1}{B}\sum_{b=1}^{B}T_{b}(x_{i})$. Readers are referred to Breiman (2001) and Waldmann (2016) for details on the theory of RF. Tree hyper-parameters, including the number of trees (ntree), number of independent variables (features) sampled in each iteration (mtry), and number of samples in the final nodes (nodesize) must be defined by the user. For **dataset** 1 we assessed the following combinations of values of ntree =(100, 300, 500), mtry =(30, 50, 100) and nodesize =(2, 5, 15), while for dataset 2, we used the same combination of values for ntree and nodesize, but a different combination of the number of feature samples, mtry=(150, 230, 320).

### Zero altered Poisson random forest

The two versions of the zero altered Poisson random forests (ZAP_RF and ZAPC_RF) like zero altered Poisson (ZAP) regression models, assumed that Y=0 with probability θ (0≤θ<1), and that Y follows a zero truncated Poisson distribution with parameter μ (μ>0), given that Y>0 (Mathlouthi *et al.* 2019). That is, they are based on the ZAP random variable:

PY=y=θ y=01-θexp⁡(-μ)μy1-exp⁡-μy! y>0.

The mean and variance for ZAP are:

$$E\left(Y\right)=\frac{\left(1-\theta \right)\exp\left(-\mu \right)}{\left(1-\exp\left(-\mu \right)\right)}\;\mathrm{and}\;\mathrm{Var}(Y)=\frac{\left(1-\theta \right)}{\left(1-\exp\left(-\mu \right)\right)}(\mu +\mu^{2})-{\left(\frac{\left(1-\theta \right)}{\left(1-\exp\left(-\mu \right)\right)}\mu \right)}^{2}.$$

In general, zero altered models are two-part models, where the first part is a logistic model, and the second part is a truncated count model. However, under the ZAP_RF and ZAPC_RF, instead of assuming a linear predictor (like ZAP regression models), it is assumed that the links between the covariates and the responses (Mathlouthi *et al.* 2019) through μ and θ are given by nonparametric link functions like:

(1) $$\log\left(\mu \right)=f_{\mu }\left(x\right)\;\mathrm{and}\;\log\left(\frac{\theta }{1-\theta }\right)=f_{\theta }\left(x,\right)$$

where $f_{\mu }$ and $f_{\theta }$ are general unknown link functions. A general nonparametric and flexible procedure can be used to estimate $f_{\mu }$ and $f_{\theta }$ in (1). However, here we used random forest in two steps instead of a parametric model:

Step 1. Zero model. Fit a binary RF to the response $I\left(Y=0\right)$, that is, the binary variable takes a value of 1 if Y=0 and a value of 0 if Y>0. This model produces estimates of $\hat{\theta }$.

Step 2. Truncated model. Fit an RF using only the positive (Y>0) observations. Assume there are $N^{+}$ such observations denoted by $Y_{1}^{+},\;\ldots ,Y_{N^{+}}^{+}$. This model produces estimates of $\hat{\mu }$. However, to exploit the Poisson assumption, the splitting criteria used in the RF with the truncated part was derived from the zero truncated Poisson likelihood that is equal to:

(2) $$LL^{+}=-N^{+}\log\left(1-\exp\left(-\mu \right)\right)+\log\left(\mu \right)\sum_{i}^{N^{+}}Y_{i}^{+}-\;N^{+}\mu -\sum_{i}^{N^{+}}\log\left(Y_{i}^{+}!\right),$$

where $LL^{+}$ is the log-likelihood function of a sample of a zero truncated Poisson distribution. The estimate of μ is obtained by solving $\frac{\partial LL^{+}}{\partial \mu }=0$, which reduces to:

$$\frac{\sum_{i}^{N^{+}}Y_{i}^{+}\;}{N^{+}}=\frac{\mu }{1-\exp(-\mu )}.$$

For a given candidate split, the loglikelihood function given in equation (2) is computed separately in the two children nodes and the best split is the one that maximizes:

$$\hat{LL^{+}}(\mathrm{left}\;\mathrm{node})\;+\hat{LL^{+}}(\mathrm{right}\;\mathrm{node}),$$

where $\hat{LL^{+}}$(left node) and $\hat{LL^{+}}($right node) are the log-likelihood for each node.

Once we have the estimates of μ and θ, the predicted values of Y under the ZAP_RF are obtained with:

$$\hat{Y}=\frac{\left(1-\hat{\theta }\right)e\mathrm{xp}(-\hat{\mu })}{\left(1-\exp\left(-\hat{\mu }\right)\right)}.$$

It is important to point out that in the prediction formula given above, ($\hat{Y}$) is equal to the mean of the ZAP model, while under the ZAPC_RF, the predictions are obtained as:

Y^=0, θ^>0.5μ^, θ^≤0.5.

The ZAPC_RF is a conventional logistic regression model where the predicted values are probabilities and those probabilities are converted to a binary outcome if the probability is larger (or smaller) than some probability threshold (most of the time this threshold is 0.5). However, under the ZAPC_RF, instead of converting the probabilities to 0 and 1, we convert to zero if $\hat{\theta }>0.5$ and to the estimated expected count value ($\hat{\mu })$ if $\hat{\theta }\leq 0.5$. One limitation of the ZAPC_RF (similar to the logistic regression) is that the probability threshold is not unique since many other values between zero and one can be used. However, the threshold value of 0.5 is used most of the time since it assumes no prior information, and for this reason, both categories have the same probability of occurring.

## Experimental data

### Phenotypic dataset 1

This dataset is composed of 115 spring wheat lines developed by the International Maize and Wheat Improvement Center (CIMMYT) and the trait measured was Fusarium head blight (FHB) severity. The experiments were performed in 2011 and data were collected in three environments (Env1, Env2, and Env3). These datasets were the same ones used by Montesinos-López *et al.* (2016) in their paper for count data with genotype × environment interaction. A full description of this dataset can be found in Montesinos-López *et al.* (2020b).

### Genotypic dataset 1

For each line under study, we used 1635 SNPs, that resulted after quality control genotyped using an Illumina 9 K SNP chip with 8632 single nucleotide polymorphisms (SNPs) (Cavanagh *et al*. 2013). Markers were coded as zero (absence) or one (presence). Specific details of this genotypic information are available in Montesinos-López *et al.* (2020b).

### Phenotypic dataset 2

In this dataset, three traits were measured *Pyrenophora tritici-repentis* (PTR), *Parastagonospora nodorum* (SN) and *Bipolaris sorokiniana* (SB) in 438 lines. The 438 wheat lines were evaluated in the greenhouse in six replicates that are considered as environments (Env1, Env2, Env3, Env4, Env5, and Env6). Therefore, the total number of observations were 438 × 6 = 2628 observations. More details of these phenotypic datasets can be found in Montesinos-López *et al.* (2020b).

### Genotypic dataset 2

In this dataset, after quality control and imputations, 11,617 SNPs were still available and these markers also were coded as zero or one. This genotypic information was used for evaluation in terms of prediction performance of the proposed models. More details of these phenotypic datasets can be found in Montesinos-López *et al.* (2020b).

### Metrics used to measure prediction performance

Cross-validation was used to evaluate the prediction performance in unseen data. Since our data contain the same lines in I environments, we used an outer fivefold cross-validation that mimics a situation where lines were evaluated in some environments for all traits but some lines were missing in other environments. We used cross-validation because the resulting test error is very nearly unbiased and because our datasets are not very large (Theodoridis 2020). Four folds were used for training and onefold for testing. We repeated the training 5 times, each time selecting one part (different each time) of the data for testing and the remaining 4 parts for training. This cross-validation strategy gives us the advantage of testing with one part of the data that has not been involved in training, so it can be considered independent, and eventually at the same time using all the data, both for training and testing (Theodoridis 2020). We reported the average prediction performance combining the 5 estimates of the testing sets in terms of average Spearman correlation (ASC), mean arctangent absolute percentage error (MAAPE) and mean absolute error of prediction (MAE), for each environment and across environments. The ASC was used instead of Pearson’s correlation because the response variable is not normally distributed. In terms of ASC, the closer to one, the better the prediction performance, while under MAE and MAAPE, the closer to zero, the better the prediction performance. It is important to point out that the process for tuning the hyper-parameter (λ) in the generalized Poisson regression (GPR) was done with 10-fold inner cross-validation, while the tuning process for the random forest models (RF, ZAP_RF, ZAPC_RF) was done with 5-fold inner cross-validation inside each outer fold. This means that in each outer fold, 20% of the data was used for tuning (TUN) and 80% of the information for inner training (ITRN). Each of the 9 (**data_set_1** and **data_set_2**) combinations of the grid search was trained with the inner training set in each outer fold; its prediction performance was evaluated in the inner tuning (TUN) set and the average in terms of MAE was obtained for each fold of the 5 inner tuning sets. For estimating the lambda hyper-parameter (λ) in GPR, we used 10-fold partition. These are the default values for the software and do not require significant amounts of computational resources, while for random forest, we used only fivefold since random forest is performed for each combination of hyper-parameters and this increases considerably the computational resources.

After selecting the best combination of hyper-parameters in terms of MAE, the model was refitted, but using the whole outer training set (80% of data) in each fold. Finally, for each outer testing set, we computed each of the three metrics (ASC, MAAPE and MAE) with its corresponding standard error (SE); then the average of the 5 outer folds and its SE was reported as a measure of prediction performance and variability in each metric. It is important to point out that the 5-fold cross-validation strategy was implemented with only 1 replication. The cv.zap.rf() function developed in the R statistical software to implement the ZAP_RF and ZAPC_RF proposed models is given in Appendix A.

#### Variable importance measures

For the proposed zero altered Poisson methods (ZAP_RF and ZAPC_RF), it was possible to obtain variable importance measures (VIM), since there are many measures of variable importance. One common approach for regression trees is to calculate the decrease in prediction accuracy from the testing dataset. For each tree, the testing set portion of the data was passed through the tree and the prediction error (PE) was recorded. Each predictor variable was then randomly permuted and j new PE were calculated. The differences between the two were then averaged over all trees, and normalized by the standard deviation of the differences. The variable showing the largest decrease in prediction accuracy was the most important variable. The results were displayed in a variable importance plot of the top ranked variables. Since the ZAP_RF and ZAPC_RF models are composed of a zero part and a truncated part, two plots were obtained for each trait, and the final VIM estimates of each independent variable were the average values of the five implemented testing sets.

### Data availability

The phenotypic and genotypic data for **dataset** 1 used in this study are contained in the R file Data_Real_Count.RData, and available at the following link: http://hdl.handle.net/11529/10575. For **dataset** 2, the phenotypic and genotypic data are contained in the R file Data_set 2.RData, available at the following link: http://hdl.handle.net/11529/10548438.

## Results

The results are given in three subsections. In the first subsection, for each trait in each dataset, we show the percentage of excess zeros. In the second subsection, we give a description of the prediction performance of **dataset** 1, while in the third subsection, the same description is given, but for **dataset** 2.

### Percentage of excess zeros in each dataset

**Figure 1:**
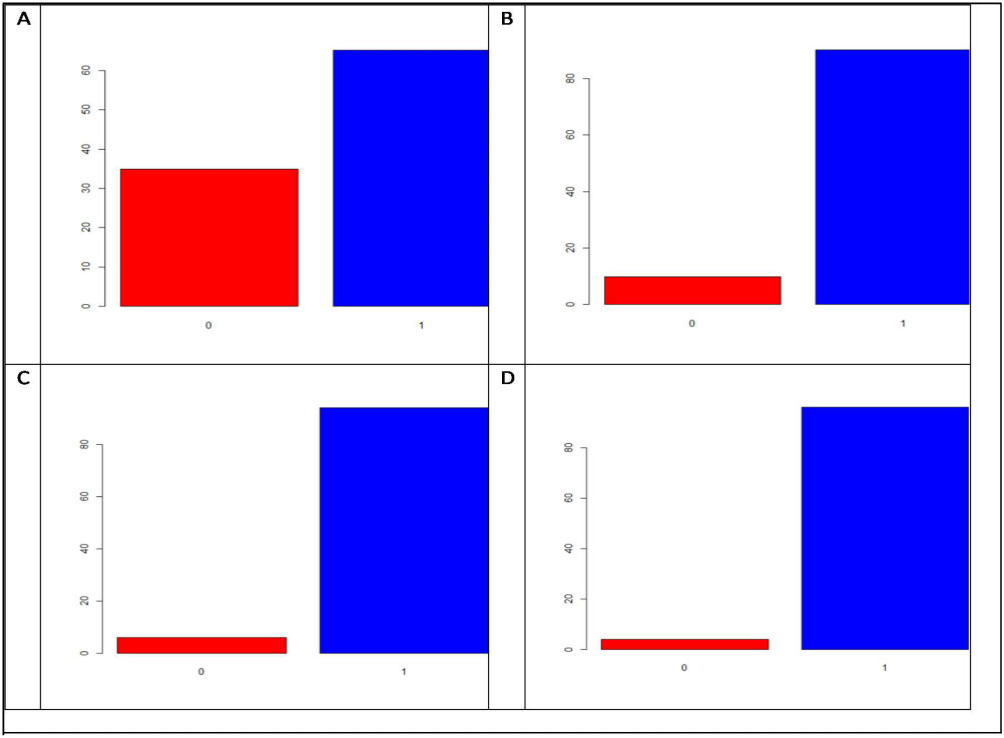
Percentages of excess zeros for each trait in **datasets 1** and **2**. Trait FHB. (A) belongs to **dataset 1**, while traits PTR (B), SB (C) and SN (D) belong to **dataset 2**.

Figure 1A shows that in trait FHB that belongs to **dataset** 1, the percentage of zeros was 34.87%. For trait PTR that belongs to **dataset** 2, the percentage of zeros was 5.97%, while for the second trait (SB) in this dataset, the percentage of zeros was 9.86% and for the last trait (SN) in this second dataset, the percentage of zeros was 3.96%. Table 1 provides a summary of the phenotypic information of each of the four traits under study, where it is evident that the mean and median are quite different, which is an indicator that the data are not symmetric and non-normally distributed. Table 1 also shows that the minimum count is zero in the four traits and the maximum is 20 in two traits (SB and SN), 18 in trait FHB and 19 in trait PTR.

**Table 1:** Summary of the phenotypic values of 4 traits under study in datasets 1 and 2

Dataset	Trait	Min.	1st Qu.	Median	Mean	3rd Qu.	Max.
1	FHB	0.000	0.000	1.000	1.780	2.000	18.000
2	PTR	0.000	4.000	5.000	6.056	9.000	19.000
2	SB	0.000	4.000	5.000	5.788	7.000	20.000
2	SN	0.000	4.000	4.000	6.284	9.000	20.000

Table 2 gives the phenotypic correlation between the environments of each trait. In trait FHB (dataset 1), we can observe a perfect correlation between environments Batan2012 and Batan2014, but a poor correlation between environment Chunchi2014 and Batan2012 and Batan2014. In trait PTR (dataset 2) the largest correlation (0.605) was between Env5 and Env6, while the lowest (0.291) was between Env3 and Env6. Most of the correlations between environments are between 0.3 and 0.4. In trait SN (dataset 2), the largest correlation (0.79) was also observed between Env5 and Env6, while the lowest (0.412) was between Env1 and Env5; the remaining correlations were between the minimum and maximum values mentioned before. Finally, for trait SB (dataset 2), the largest (0.456) and minimum (0.316) correlations were between Env1 and Env2 and between Env2 and Env4, respectively.

**Table 2:** Phenotypic correlation between environments in each trait (FHB of **dataset 1** and PTR, SN and SB of **dataset 2**)

		Trait=	FHB			
		Batan 2012	Batan 2014	Chunchi 2014		
	Batan 2012	**1.000**	**1.000**	0.042		
	Batan 2014	**1.000**	**1.000**	0.042		
	Chunchi 2014	0.042	0.042	1.000		

		**Trait=**	**PTR**			
	Env1	Env2	Env3	Env4	Env5	Env6
Env1	1.000	0.449	0.383	0.333	0.364	0.351
Env2	0.449	1.000	0.339	0.308	0.357	0.317
Env3	0.383	0.339	1.000	0.546	0.293	**0.291**
Env4	0.333	0.308	0.546	1.000	0.294	0.325
Env5	0.364	0.357	0.293	0.294	1.000	**0.605**
Env6	0.351	0.317	0.291	0.325	0.605	1.000

		**Trait=**	**SN**			
	Env1	Env2	Env3	Env4	Env5	Env6
Env1	1.000	0.616	0.534	0.608	**0.412**	0.475
Env2	0.616	1.000	0.509	0.624	0.456	0.471
Env3	0.534	0.509	1.000	0.711	0.472	0.486
Env4	0.608	0.624	0.711	1.000	0.487	0.510
Env5	0.412	0.456	0.472	0.487	1.000	**0.790**
Env6	0.475	0.471	0.486	0.510	0.790	1.000

		**Trait=**	**SB**			
	Env1	Env2	Env3	Env4	Env5	Env6
Env1	1.000	**0.456**	0.433	0.362	0.400	0.366
Env2	0.456	1.000	0.437	**0.316**	0.397	0.363
Env3	0.433	0.437	1.000	0.419	0.455	0.442
Env4	0.362	0.316	0.419	1.000	0.371	0.399
Env5	0.400	0.397	0.455	0.371	1.000	0.442
Env6	0.366	0.363	0.442	0.399	0.442	1.000

The largest and smallest correlations are in bold.

### Dataset 1

**Figure 2:**
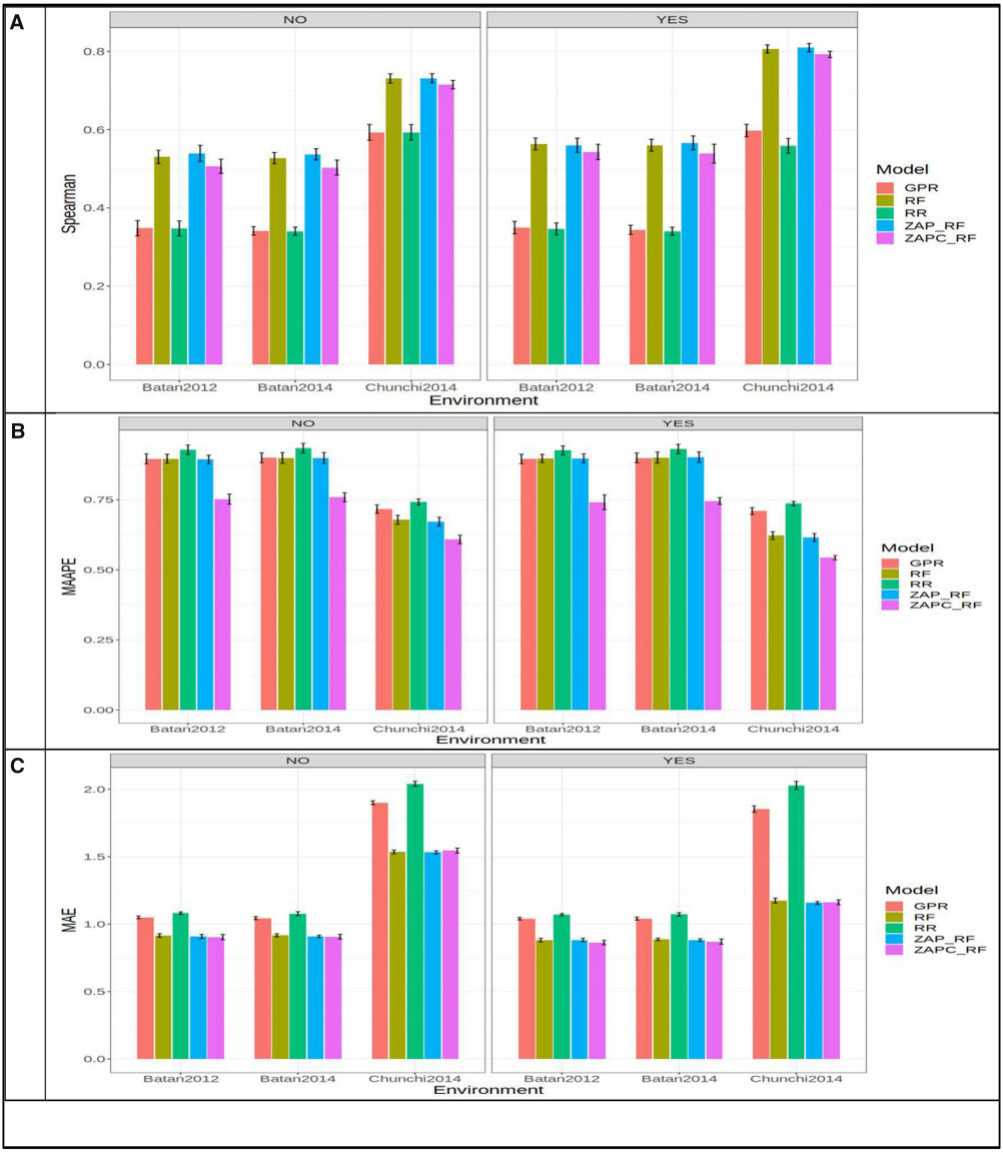
Prediction performance in terms of average Spearman’s correlation (Spearman; A), mean arctangent absolute percentage error (MAAPE; B) and mean absolute error of prediction (MAE; C) of the five models (GPR, RF, RR, ZAP_RF, ZAPC_RF) for each environment in **dataset 1** for trait FHB. NO in the plots means that the genotype×environment (GE) interaction was ignored, while YES means that the GE interaction term was taken into account.

In Figure 2, we compare the prediction performance of the five models (GPR, RF, RR, ZAP_RF, ZAPC_RF) in **dataset** 1 for trait FHB. The prediction performance was evaluated in terms of Spearman’s correlation, MAAPE and MAE for each environment. First we provide the results taking into account the genotype by environment (GE) interaction in the predictor. In terms of ASC, Figure 2A shows that the best prediction performance (in the three environments) was observed under the ZAP_RF model in environment Chunchi2014, while the worst was observed under the RR model in environment Batan2014, and the best model outperformed the worst by $\left(0.809\;-\;0.340\right)\times \frac{100}{0.809}=57.920\%$. In each environment, the ZAP_RF model outperformed the RR by $\left(0.560\;-\;0.346\right)\times \frac{100}{0.560}=38.160\%$ (in Batan2012), $\left(0.566\;-\;0.341\right)\times \frac{100}{0.566}=39.87\%$ (in Batan 2014) and by $\left(0.809\;-\;0.559\right)\times \frac{100}{0.809}=30.97\%$ (in Chunchi 2014). In MAAPE terms, Figure 2B shows that in the three environments, the best performance was under the ZAPC_RF model and the worst was under the RR model. In Batan2012, Batan2014, and Chunchi2014, the ZAPC_RF outperformed the RR model by $\left(0.927\;-\;0.74\right)\times \frac{100}{0.74}=25.270\%$, $\left(0.932\;-\;0.748\right)\times \frac{100}{0.748}=24.599\%$ and $\left(0.737\;-\;0.544\right)\times \frac{100}{0.544}=35.478\%$, respectively. The second best model was ZAP_RF, which was slightly better than the RF model, but there were no significant differences between them in terms of MAAPE performance (Figure 2B). In MAE terms, the ZAPC_RF model was also the best in environments Batan2012 and Batan2014, while in environment Chunchi2014, the best model was RF (Figure 2C). In environments Batan2012 and Batan2014, ZAPC_RF outperformed the worst model (RR) by $\left(1.072\;-\;0.863\right)\times \frac{100}{0.863}=24.218\%$ and $\left(1.074\;-\;0.872\right)\times \frac{100}{0.872}=23.165\%$, respectively, while in environment Chunchi2014, the best model ZAP_RF outperformed the worst model RR by $\left(2.029\;-\;1.159\right)\times \frac{100}{1.159}=75.065\%$. Without the GE interaction term, we can see that in terms of Spearman’s correlation, the best model was ZAP_RF (Spearman = 0.731; Chunchi2014) and the worst was model RR (Spearman = 0.341; Batan2014), and the ZAP_RF outperformed the RR model by $\left(0.731\;-\;0.341\right)\times \frac{100}{0.731}=53.43\%$ (Figure 2A). In terms of MAAPE, the best and worst models were ZAPC_RF (MAAPE = 0.608; Chunchi2014) and RR (MAAPE = 0.934; Batan2014) and the best model outperformed the worst by $\left(0.934\;-\;0.608\right)\times \frac{100}{0.608}=53.52\%$ (Figure 2B). Finally, in terms of MAE, the best model (ZAPC_RF) outperformed the worst by $\left(2.0412\;-\;0.904\right)\times \frac{100}{0.904}=125.83\%$ (Figure 2C).

**Figure 3:**
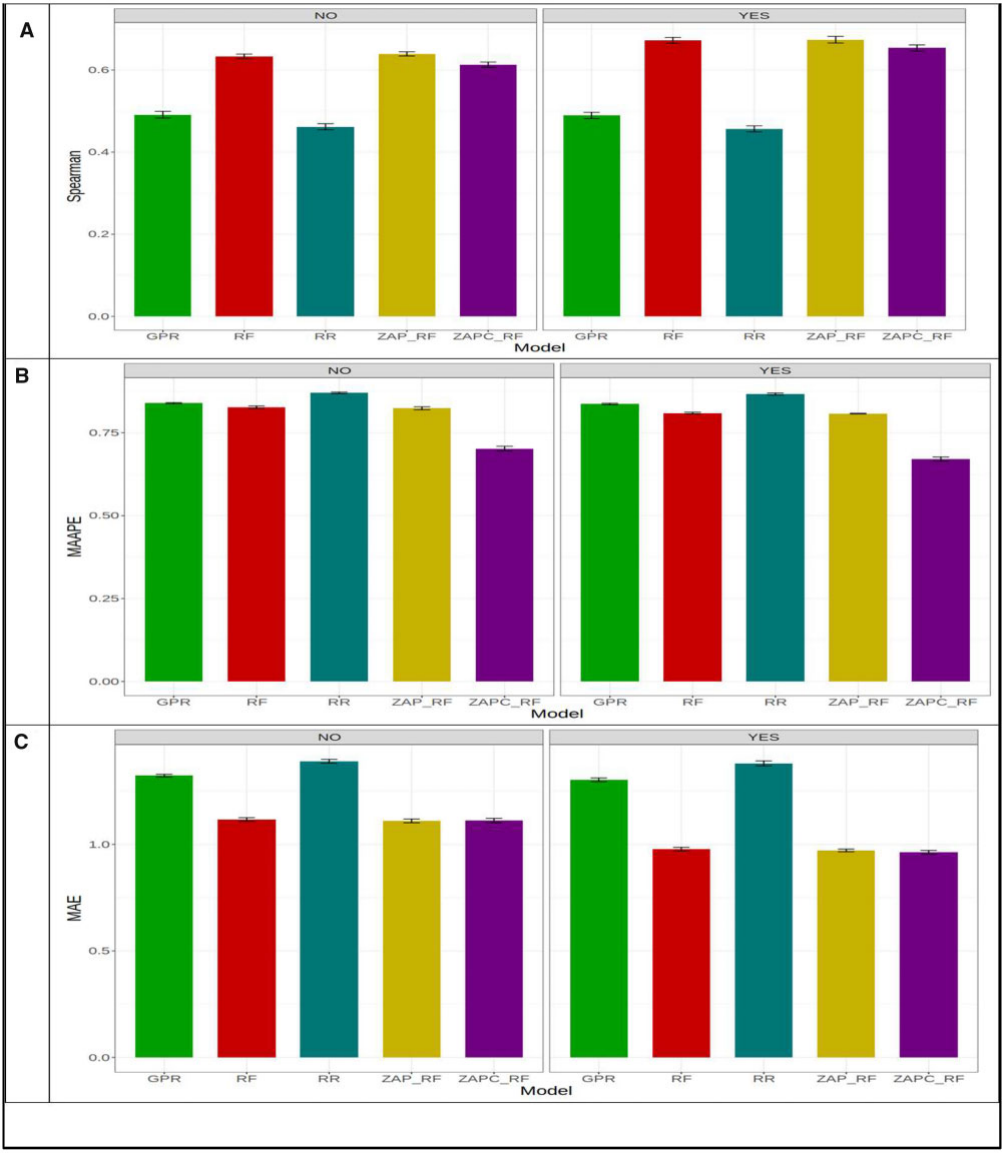
Prediction performance in terms of average Spearman’s correlation (Spearman; A), mean arctangent absolute percentage error (MAAPE; B) and mean absolute error of prediction (MAE; C) of the five models (GPR, RF, RR, ZAP_RF, ZAPC_RF) across environments in **dataset 1** for trait FHB. NO in the plots means that the genotype×environment (GE) interaction was ignored, while YES means that the GE interaction term was taken into account.

Figure 3A indicates that under Spearman’s correlation across environments, the best and worst models were ZAP_RF and RR, respectively. The best model outperformed the worst model by $\left(0.674\;-\;0.456\right)\times \frac{100}{0.674}=32.22\%$. Across environments, Figure 3B shows that in MAAPE terms, the ZAPC_RF model was the best and the RR model was the worst. The ZAPC_RF model outperformed the worst model (RR) by $\left(0.867\;-\;0.671\right)\times \frac{100}{0.671}=29.210\%$. The second best model in terms of MAAPE was the ZAP_RF, which was outperformed by the ZAPC_RF by $\left(0.808\;-\;0.671\right)\times \frac{100}{0.671}=20.417\%$. In terms of MAE, the best model was also ZAPC_RF, which outperformed the worst model RR by $\left(1.381\;-\;0.964\right)\times \frac{100}{0.964}=43.257\%$. In terms of MAE, the ZAP_RF model was also the second best and was outperformed by the best model (ZAPC_RF) by only $\left(0.972\;-\;0.964\right)\times \frac{100}{0.964}=0.83\%$ (Figure 3B). Without GE interaction across environments, the best and worst models in terms of Spearman’s correlation were ZAP_RF (Spearman = 0.639) and RR (Spearman = 0.461), respectively, where the first model outperformed the worst model by $\left(0.639\;-\;0.461\right)\times \frac{100}{0.639}=27.75\%$. In terms of MAAPE, the best and worst models were ZAPC_RF (MAAPE = 0.702) and RR (MAAPE = 0.870), respectively, where ZAPC_RF outperformed the RR model by $\left(0.870\;-\;0.702\right)\times \frac{100}{0.702}=23.94\%$. Finally, in terms of MAE, the best model (ZAP_RF, MAE = 1.11) outperformed the worst model (RR; MAE = 1.389) by $\left(1.389\;-\;1.110\right)\times \frac{100}{1.110}=25.17\%$.

**Figure 4:**
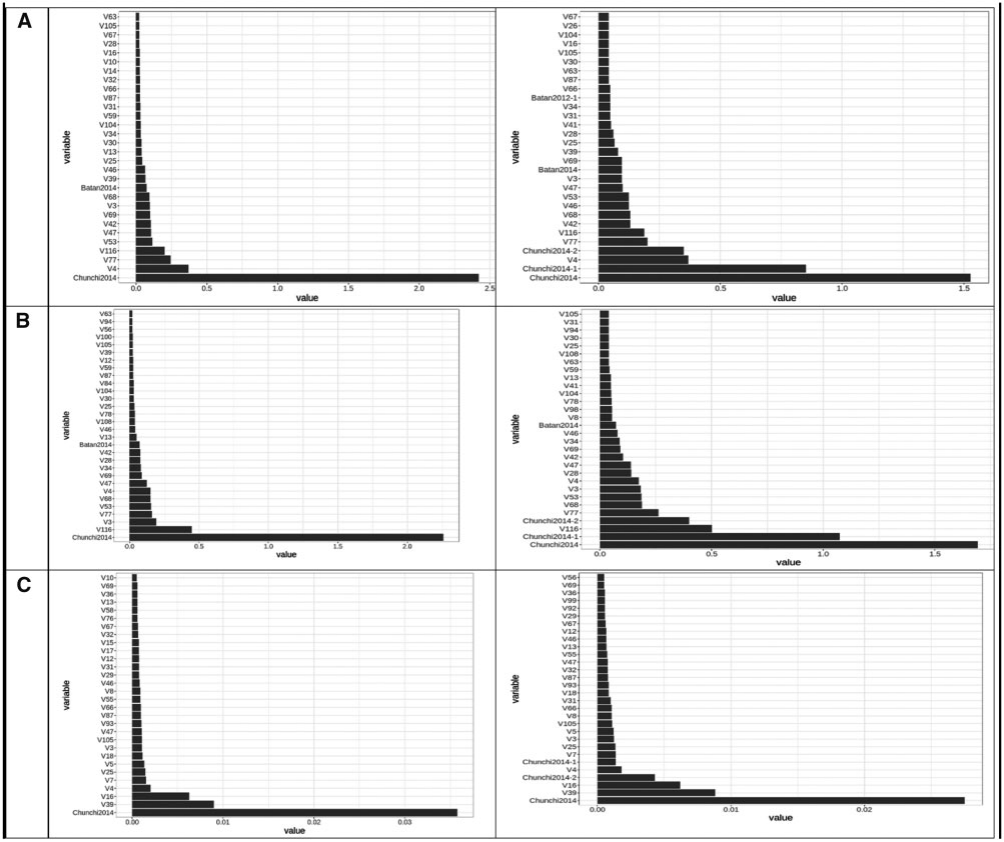
Predictor importance for trait FHB in **dataset 1** under conventional random forest (A) and under zero altered Poisson random forest (B and C). The first column contains the results without interaction (NO) and the second column contains the results with interaction (YES).

Figure 4 provides the variable important values (VIM) for the conventional random forest model (A) and for the ZAP_RF model (B, C). Figure 4B corresponds to the truncated part (**A**) and Figure 4C to the zero part (**B**) of the ZAP_RF model. These plots only contain the 30 most important variable important measures. Figure 4A indicates that the three most important predictors for the conventional RF model are Chunchi2014, V4, and V77 (without GE interaction) and Chunchi2014, Chunchi2014-1 and V4 (with GE interaction), while under the ZAP_RF model, the three most important predictors, under the truncated part ignoring the GE interaction, were Chunchi2014, V116 and V3, while taking into account the GE interaction term were Chunchi2014, Chunchi2014-1 and V116. Under the zero part, predictors Chunchi2014, V39, V16 (without the GE interaction) and predictors Chunchi2014, V39 and V16 are the most important predictors taking into account the GE interaction term.

### Dataset 2

#### Trait PTR

**Figure 5:**
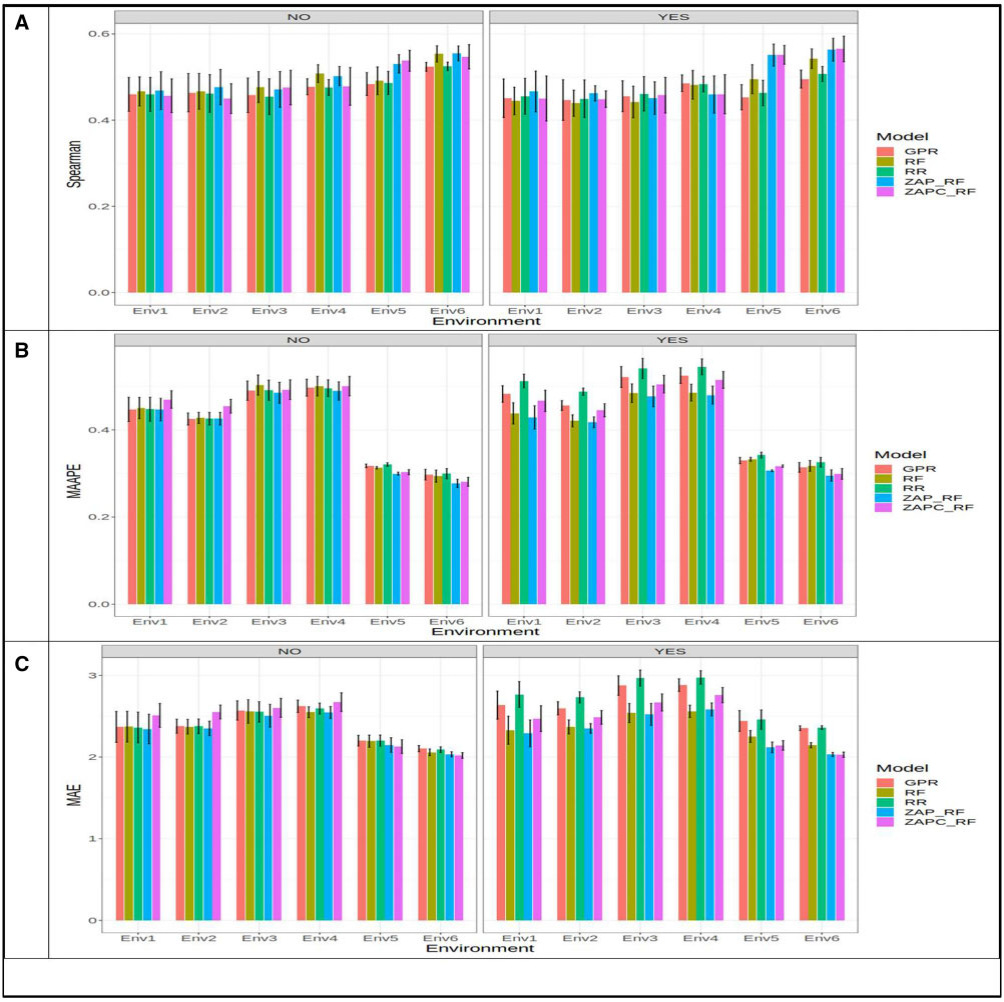
Prediction performance in terms of average Spearman’s correlation (Spearman; A), mean arctangent absolute percentage error (MAAPE; A) and mean absolute error of prediction (MAE; C) of the five models (GPR, RF, RR, ZAP_RF, ZAPC_RF) for each environment in **dataset 2** for trait PTR. NO in the plots means that the genotype×environment (GE) interaction was ignored, while YES means that the GE interaction term was taken into account.

First, we give the results for trait PTR, then for trait SB and finally for SN. The prediction performance of the five models (GPR, RF, RR, ZAP_RF, ZAPC_RF) of **dataset** 2 for trait PTR was evaluated in terms of Spearman’s correlation, MAAPE and MAE for each environment (Figure 5). First, we provide the prediction performance taking into account the GE interaction. In terms of Spearman’s correlation, for the PTR trait the best and worst prediction performances were observed under the ZAPC_RF (Spearman = 0.565; Env6) and RF (Spearman = 0.439, Env2) models, respectively. The best model outperformed the worst model by $\left(0.565\;-\;0.439\right)\times \frac{100}{0.565}=22.27\%$ (Figure 5A). Under the MAAPE, the best performance was observed under the ZAP_RF model (MAAPE = 0.2953, Env6) and the worst under the RR model (MAAPE= 0.544, Env4). The ZAP_RF outperformed the RR model by $\left(0.554\;-\;0.2953\right)\times \frac{100}{0.2953}=84.49\%$ (Figure 5B), while under the MAE, the best model was the ZAPC_RF (MAE = 2.029, Env6), and the worst was RR model (MAE = 2.975, Env4), and the best model outperformed the worst by $\left(2.975\;-\;2.029\right)\times \frac{100}{2.029}=46.60\%$ (Figure 5C). Without taking into account the GE interaction, we can see that in terms of Spearman’s correlation, the best model was the ZAP_RF (Spearman = 0.554, Env6), while the worst was ZAPC_RF (Spearman = 0.450, Env2); the ZAP_RF outperformed the ZAPC_RF by $\left(0.554\;-\;0.450\right)\times \frac{100}{0.554}=18.86\%$. In terms of MAAPE, the best model was ZAP_RF (MAAPE = 0.277, Env6), while the worst was the RF (MAAPE = 0.502, Env3) model; the best model outperformed the worst by $\left(0.502\;-\;0.277\right)\times \frac{100}{0.227}=81.42\%$. In terms of MAE, the ZAPC_RF (MAE = 2.019, Env6) model was the best, while the worst model was also de ZAPC_RF(MAE = 2.672), but in environment (Env4) the best prediction of ZAPC_RF in Env6 outperformed the ZAPC_RF in Env4 by $\left(2.672\;-\;2.019\right)\times \frac{100}{2.019}=32.33\%$.

**Figure 6:**
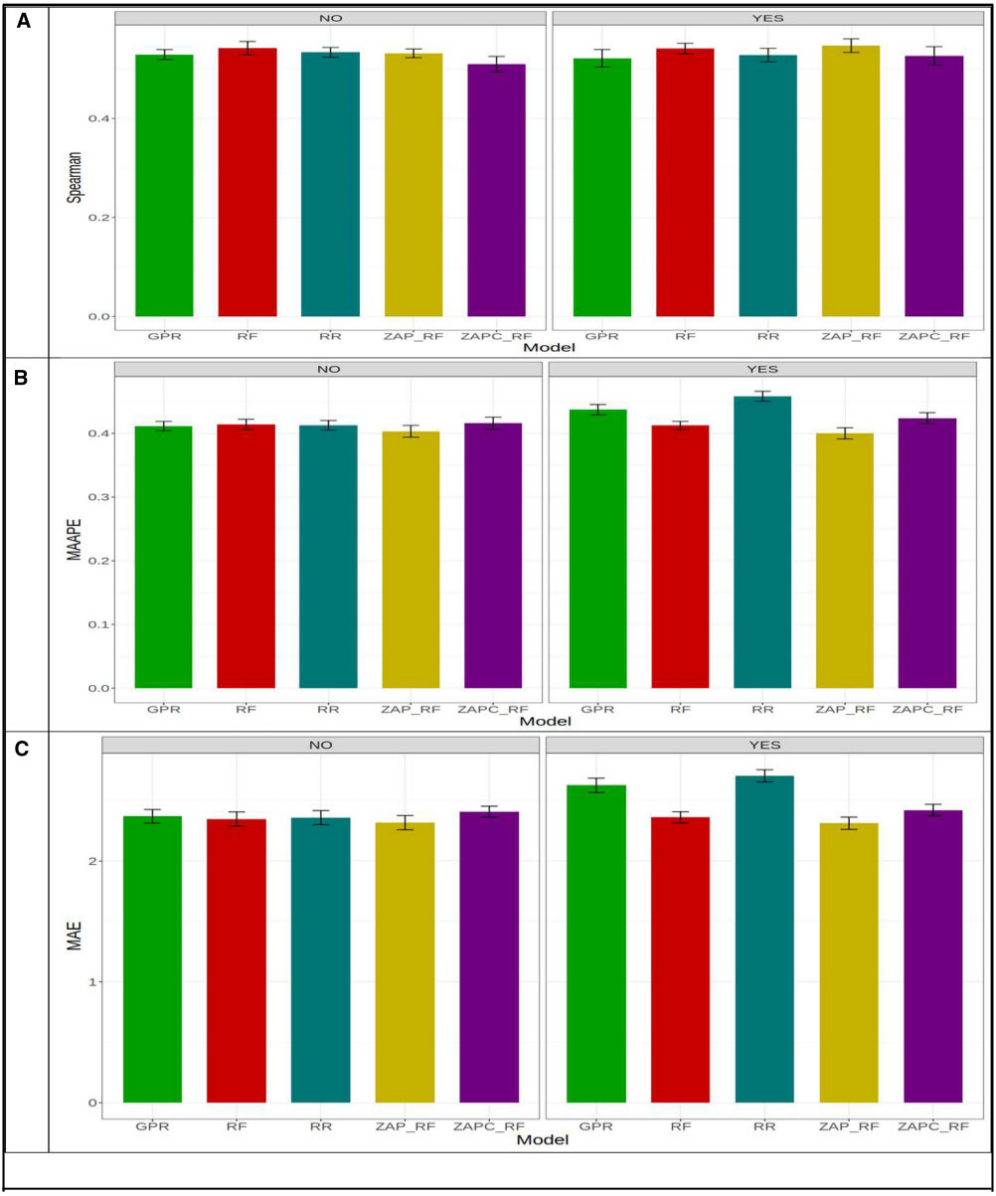
Prediction performance in terms of average Spearman’s correlation (Spearman; A), mean arctangent absolute percentage error (MAAPE; B) and mean absolute error of prediction (MAE; C) of the five models (GPR, RF, RR, ZAP_RF, ZAPC_RF) across environments in **dataset 2** for trait PTR. NO in the plots means that the genotype×environment (GE) interaction was ignored, while YES means that the GE interaction term was taken into account.

Figure 6A indicates that across-environments taking into account the GE interaction the best model was ZAP_RF (Spearman = 0.547), the worst was GPR (Spearman = 0.521) model, and the best model outperformed the worst by $\left(0.547\;-\;0.521\right)\times \frac{100}{0.547}=4.67\%$. Figure 6B shows that in terms of MAAPE, across environments, the ZAP_RF (MAAPE = 0.400) model was the best and the worst was the RR (MAAPE = 0.458) model. But the best model outperformed the worst model by only $\left(0.458\;-\;0.400\right)\times \frac{100}{0.400}=12.70\%$. In terms of MAE, the best model was also ZAP_RF (MAE= 2.312) which outperformed the worst model RR (MAE = 2.706) by $\left(2.706\;-\;2.312\right)\times \frac{100}{2.312}=14.54\%$ (Figure 6C). Without taking into account the GE interaction, the best prediction performance across-environments under Spearman’s correlation was with model RF (Spearman = 0.542) and the worst was with model ZAPC_RF (Spearman = 0.51) and model RF outperformed ZAPC_RF by $\left(0.542\;-\;0.51\right)\times \frac{100}{0.542}=5.92\%$. But, in terms of MAAPE, the best model was ZAP_RF (MAAPE = 0.403) and the worst was ZAPC_RF (MAAPE = 0.451) and the ZAP_RF outperformed the ZAPC_RF by $\left(0.451\;-\;0.403\right)\times \frac{100}{0.403}=3.17\%$. Finally, in terms of MAE, the best model (ZAP_RF) outperformed the worst model (ZAPC_RF) by $\left(2.409\;-\;2.317\right)\times \frac{100}{2.317}=3.94\%.$

**Figure 7:**
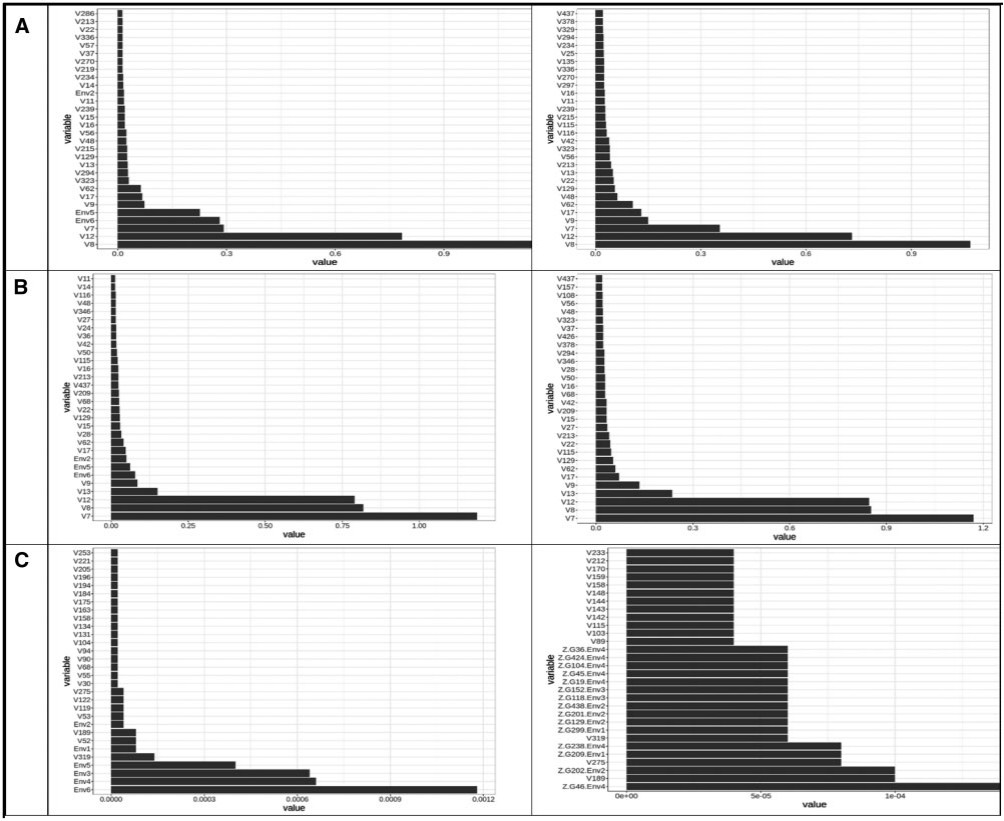
Predictor importance for trait PTR in **dataset 2** under conventional random forest (A) and under zero altered Poisson random forest for trait PTR (B and C). The first column contains the results without interaction (NO) and the second column contains the results with interaction (YES).

Figure 7 provides the VIM for the conventional random forest model (A) and for the ZAP_RF model (B, truncated part; C, zero part) for trait PTR of **dataset** 2. These plots only contain the 30 most important VIM. The three most important predictors for the conventional RF model correspond to predictors V8, V12 and V7 (with and without GE interaction), respectively. For the ZAP_RF model under the truncated part, the same predictors, V7, V8 and V12 (with and without GE interaction) were the most important predictors. Under the zero part, three dummies out of the six environments (Env6, Env4 and Env3) were the most important predictors ignoring the GE interaction, while with the GE interaction, the most important predictors were Z.G46.Env4, V189 and Z.G202.Env2.

#### Trait SB:

**Figure 8:**
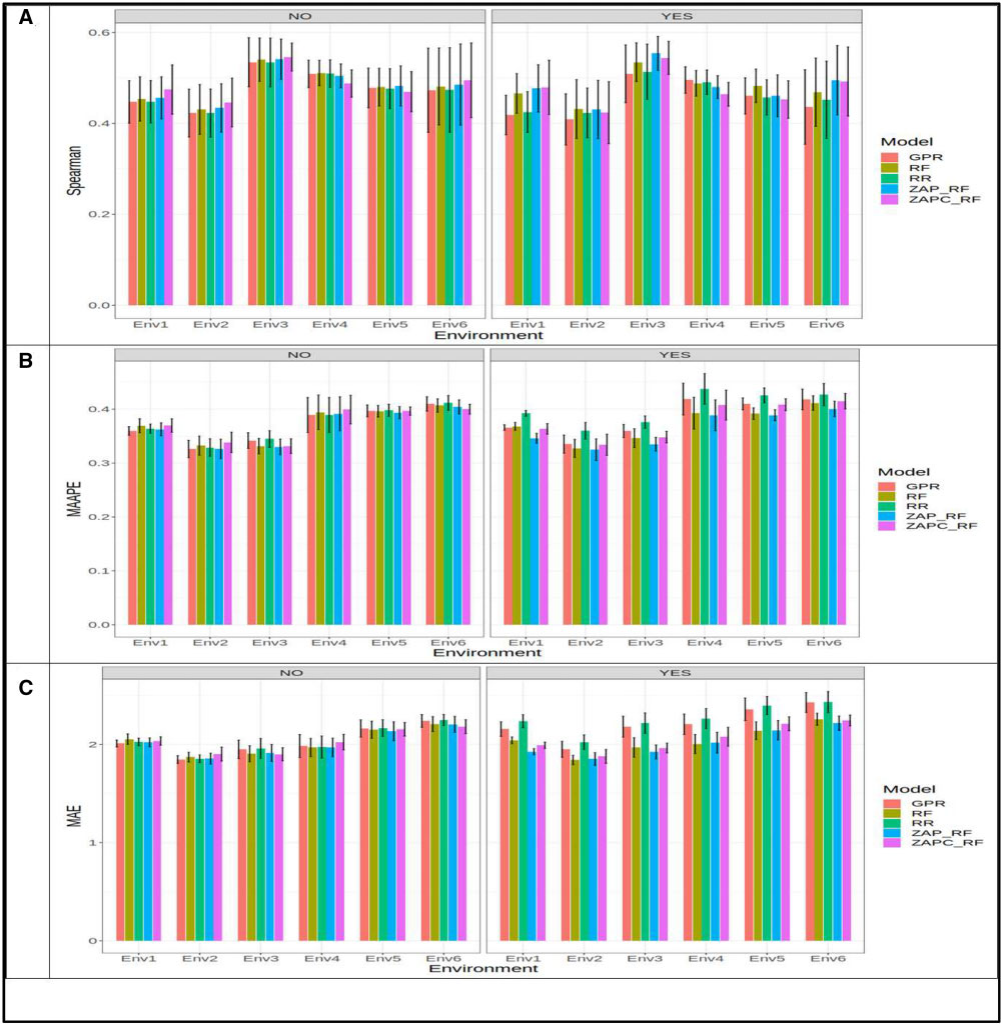
Prediction performance in terms of average Spearman’s correlation (Spearman; A), mean arctangent absolute percentage error (MAAPE; B) and mean absolute error of prediction (MAE; C) of the five models (GPR, RF, RR, ZAP_RF, ZAPC_RF) for each environment in **dataset 2** for trait SB. NO in the plots means that the genotype×environment (GE) interaction was ignored, while YES means that the GE interaction term was taken into account.

Next, we provide the results for the SB trait. The prediction performance of the five models (GPR, RF, RR, ZAP_RF, ZAPC_RF) for this trait in **dataset** 2 is reported in terms of Spearman’s correlation, MAAPE and MAE for each environment (Figure 8). First, we provide the prediction performance with the GE interaction. In terms of Spearman’s correlation, the best and worst prediction performances, in the SB trait, were observed under models ZAP_RF (Spearman = 0.554; Env3) and GPR (Spearman = 0.409, Env2), respectively. The ZAP_RF model outperformed the GPR model by $\left(0.554\;-\;0.409\right)\times \frac{100}{0.554}=26.22\%$ (Figure 8A). In terms of MAAPE, the best model was also ZAP_RF (MAAPE = 0.325; Env2), but now the worst was RR model (MAAPE = 0.437, Env4) and the ZAP_RF outperformed the RR model by $\left(0.437\;-\;0.325\right)\times \frac{100}{0.325}=34.67\%$ (Figure 8B). Under the MAE, the best model was RF (MAE = 1.842, Env2) and the worst was model RR (MAE = 2.432, Env6), and the RF model outperformed the RR by $\left(2.432\;-\;1.842\right)\times \frac{100}{2.432}=32.45\%$ (Figure 8C). Ignoring the GE interaction, in terms of Spearman’s correlation the best model was ZAPC_RF (Spearman = 0.546, Env3), while the worst was RR (Spearman = 0.423, Env2) and the ZAPC_RF outperformed the RR by $\left(0.546\;-\;0.423\right)\times \frac{100}{0.546}=22.58\%$ (Figure 8A). With MAAPE, the best models were ZAP_RF (MAAPE = 0.326, Env2) and GPR (MAAPE = 0.326, Env2), while the worst was RR (MAAPE = 0.411, Env6) and the best model outperformed the worst by $\left(0.411\;-\;0.326\right)\times \frac{100}{0.326}=26.20\%$ (Figure 8B). In terms of MAE, the GPR (MAE = 1.847, Env2) model was the best, while the worst model was the RR (MAE = 2.249, Env6) and model GPR outperformed model RR by $\left(2.249\;-\;1.847\right)\times \frac{100}{1.847}=21.79\%$ (Figure 8C).

**Figure 9:**
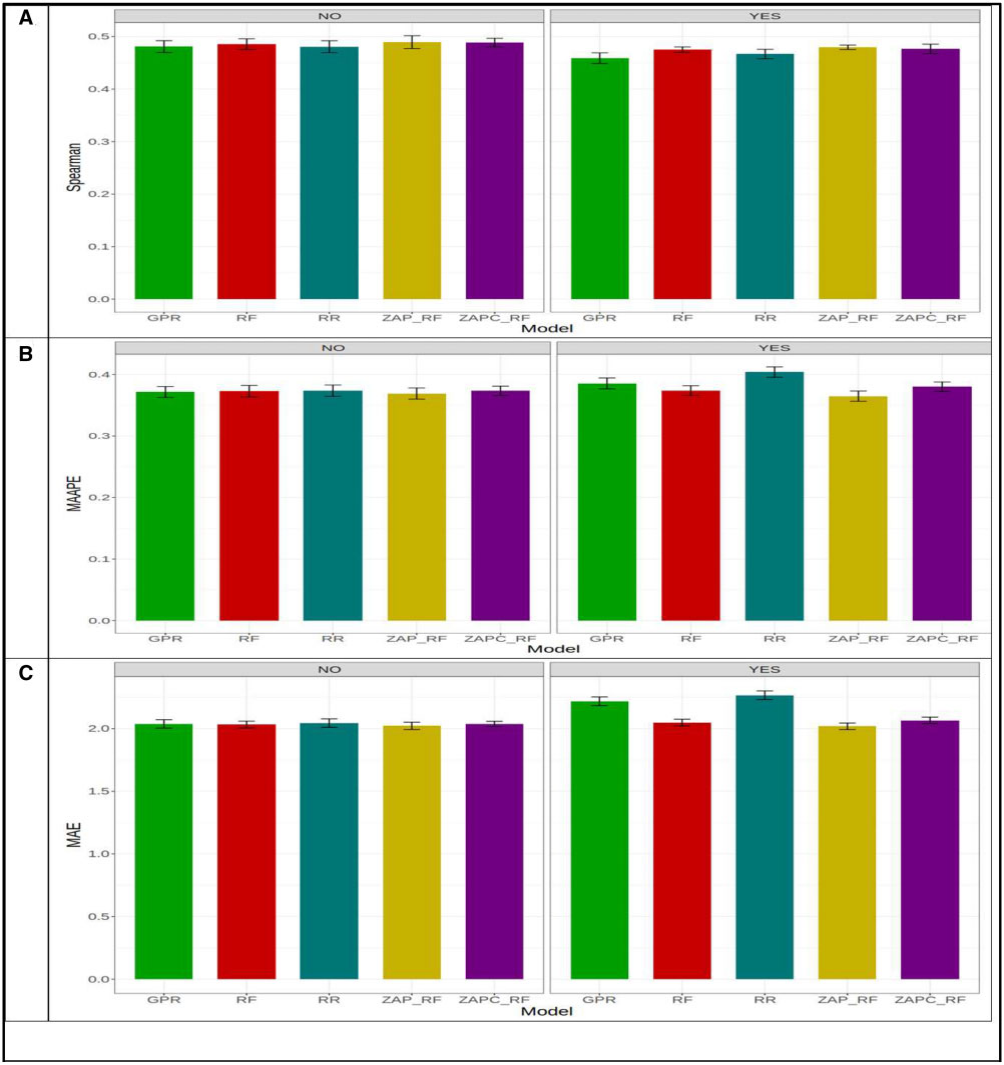
Prediction performance in terms of average Spearman’s correlation (Spearman; A), mean arctangent absolute percentage error (MAAPE; B) and mean absolute error of prediction (MAE; C) of the five models (GPR, RF, RR, ZAP_RF, ZAPC_RF) across environments in **dataset 2** for trait SB. NO in the plots means that the genotype×environment (GE) interaction was ignored, while YES means that the GE interaction term was taken into account.

In Figure 9A across-environments, taking into account the GE interaction, the best model was ZAP_RF (Spearman = 0.479) and the worst was model GPR (Spearman = 0.458) and the ZAP_RF model outperformed the GPR by $\left(0.479\;-\;0.458\right)\times \frac{100}{0.479}=4.31\%$. In terms of MAAPE (Figure 9B), across environments, the ZAP_RF (MAAPE = 0.364) model was the best and the worst was the RR (MAAPE = 0.404) model and the ZAP_RF model outperformed the RR model by $\left(0.404\;-\;0.364\right)\times \frac{100}{0.364}=10.71\%$. Under MAE, the ZAP_RF (best model with MAE = 2.018) outperformed the RR (worst model with MAE = 2.266) by $\left(2.266\;-\;2.018\right)\times \frac{100}{2.018}=12.27\%$ (Figure 9C). Without the GE interaction, in terms of Spearman’s correlation across-environments, ZAP_RF (Spearman = 0.489) was the best model and RR (Spearman = 0.480) was the worst model, and ZAP_RF outperformed the RR model by $\left(0.489\;-\;0.480\right)\times \frac{100}{0.489}=1.77\%$ (Figure 9A). Under MAAPE, ZAP_RF (MAAPE = 0.369) was the best model and RR (MAAPE = 0.373) was the worst model, and ZAP_RF outperformed the RR model by $\left(0.373\;-\;0.369\right)\times \frac{100}{0.369}=1.27\%$ (Figure 9B). Finally, in terms of MAE, ZAP_RF (the best model) outperformed the RR (worst model) by $\left(2.021\;-\;2.043\right)\times \frac{100}{2.043}=1.03\%\;($Figure 9C).

**Figure 10:**
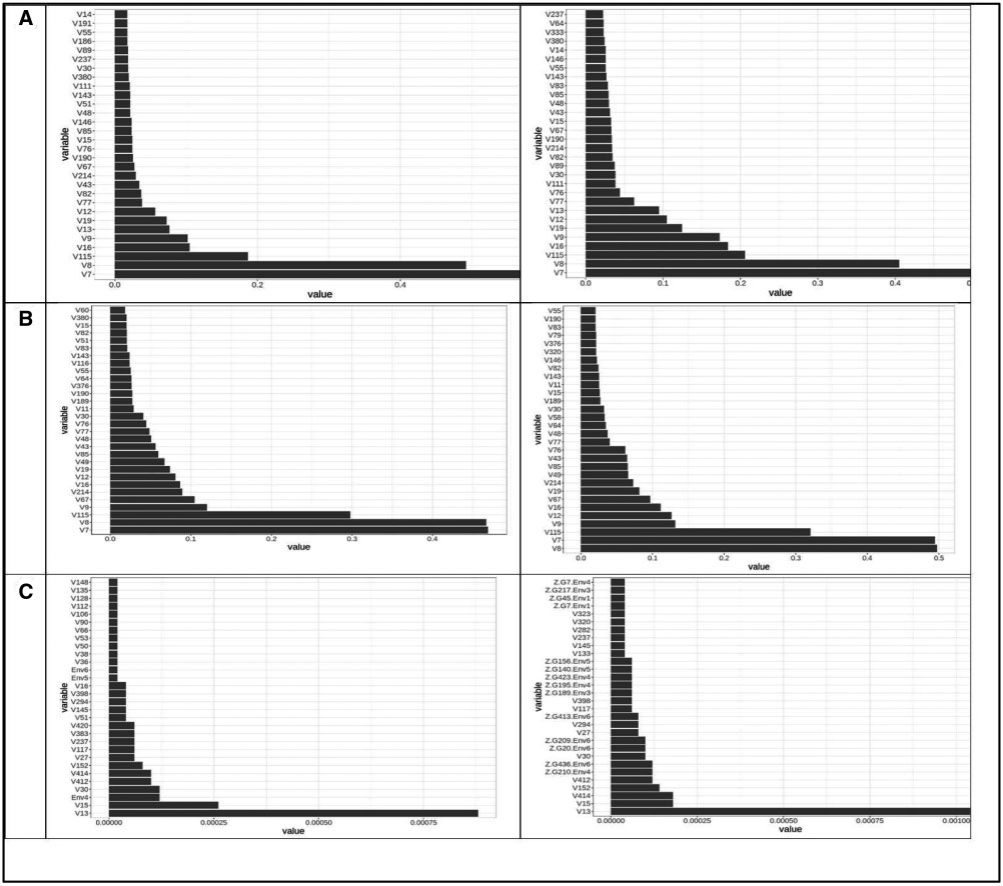
Predictor importance for trait SB in **dataset 2** under conventional random forest (A) and under zero altered Poisson random forest for trait SB (B and C). The first column contains the results without interaction (NO) and the second column contains the results with interaction (YES).

Figure 10 for the conventional random forest model (A) and for the ZAP_RF model (B, truncated part; C, zero part) for trait SB in **dataset** 2 provides the VIM. Only the 30 most important VIM are given in these plots. With and without GE interaction, the three most important predictors for the conventional RF model were V7, V8 and V115, respectively. For the ZAP_RF model under the truncated part, with and without GE interaction, the most important predictors were the same: V7, V8, and V115. Under the zero part, V13, V15 and Env4 were the most important predictors ignoring the GE interaction, while with the GE interaction the most important predictors were V13, V15, and V414.

#### Trait SN:

**Figure 11:**
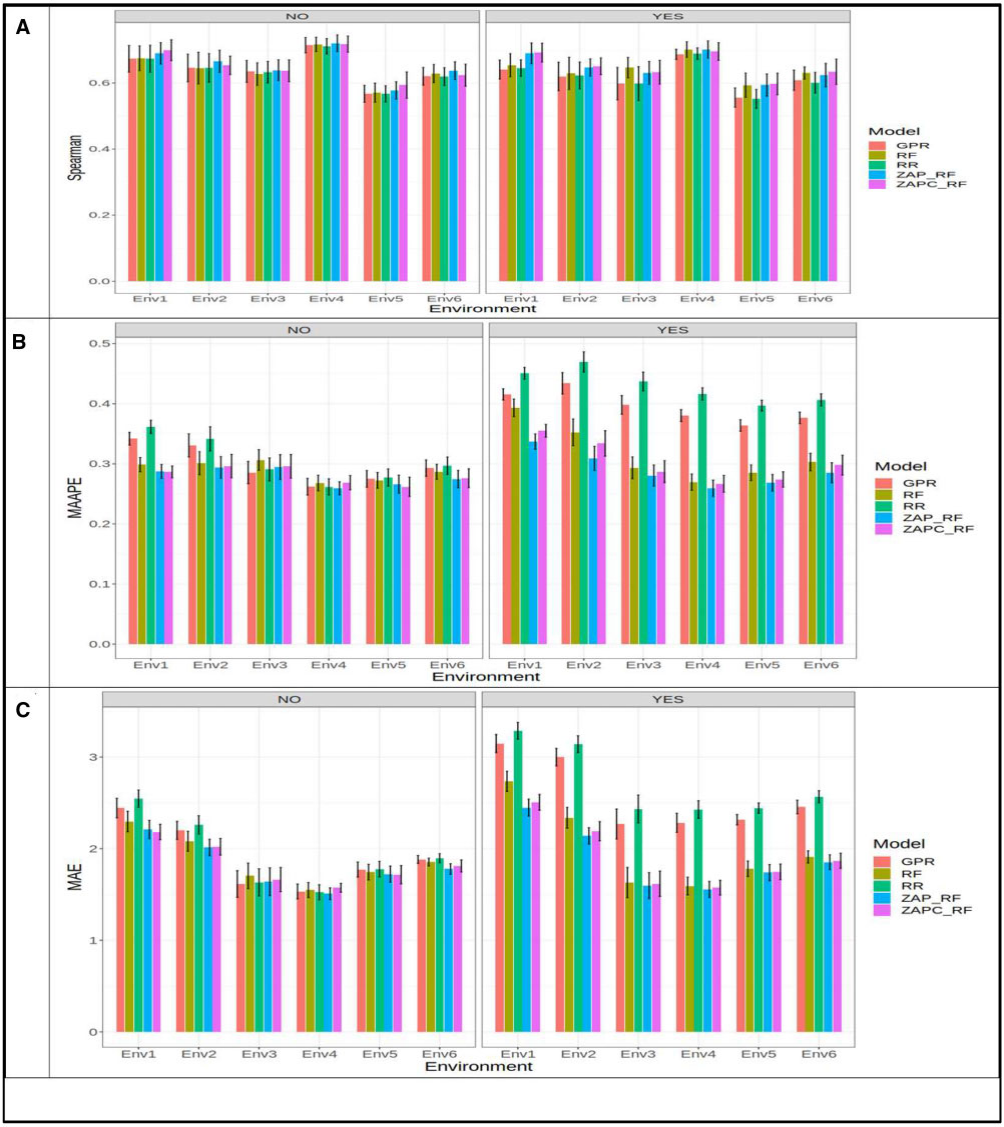
Prediction performance in terms of average Spearman’s correlation (Spearman; A), mean arctangent absolute percentage error (MAAPE; B) and mean absolute error of prediction (MAE; C) of the five models (GPR, RF, RR, ZAP_RF, ZAPC_RF) for each environment in **dataset 2** for trait SN. NO in the plots means that the genotype×environment (GE) interaction was ignored, while YES means that the GE interaction term was taken into account.

With the GE interaction term in the predictor, we can observe that in terms of Spearman’s correlation, the best model was ZAP_RF (Spearman = 0.701, Env4) and the worst was RR (Spearman = 0.552, Env5) and model ZAP_RF outperformed the RR model by $\left(0.701\;-\;0.552\right)\times \frac{100}{0.701}=21.28\%$ (Figure 11A), while in terms of MAAPE, the best model (ZAP_RF with MAAPE = 0.259 in Env4) outperformed the worst model (RR with MAAPE = 0.469 in Env2) by $\left(0.469\;-\;0.259\right)\times \frac{100}{0.259}=81.25\%$ (Figure 11B). In terms of MAE, the best model was also ZAP_RF (MAE = 1.555, Env4) and the worst was also model RR (MAE = 3.287, Env1), and model ZAP_RF outperformed the RR model by $\left(3.287\;-\;1.555\right)\times \frac{100}{1.555}=111.36\%$ (Figure 11C). Ignoring the GE interaction term, we can observe that in terms of Spearman’s correlation the best model was ZAP_RF (Spearman = 0.720, Env4) and the worst was model RR (Spearman = 0.567, Env5) and the gain of the best model over the worst model was $\left(0.720\;-\;0.567\right)\times \frac{100}{0.720}=21.27\%$ (Figure 11A). In terms of MAAPE, the best and worst models were ZAP_RF (MAAPE = 0.259, Env4) and RR (MAAPE = 0.361, Env1), and the ZAP_RF outperformed the RR by $\left(0.361\;-\;0.259\right)\times \frac{100}{0.259}=39.41\%$ (Figure 11B). Finally, in terms of MAE, the ZAP_RF (MAE = 1.508, Env4) and RR (MAE = 2.546, Env1) models were also the best and worst, respectively, and the best outperformed the worst by $\left(2.546\;-\;1.508\right)\times \frac{100}{1.508}=68.81\%$ (Figure 11C).

**Figure 12:**
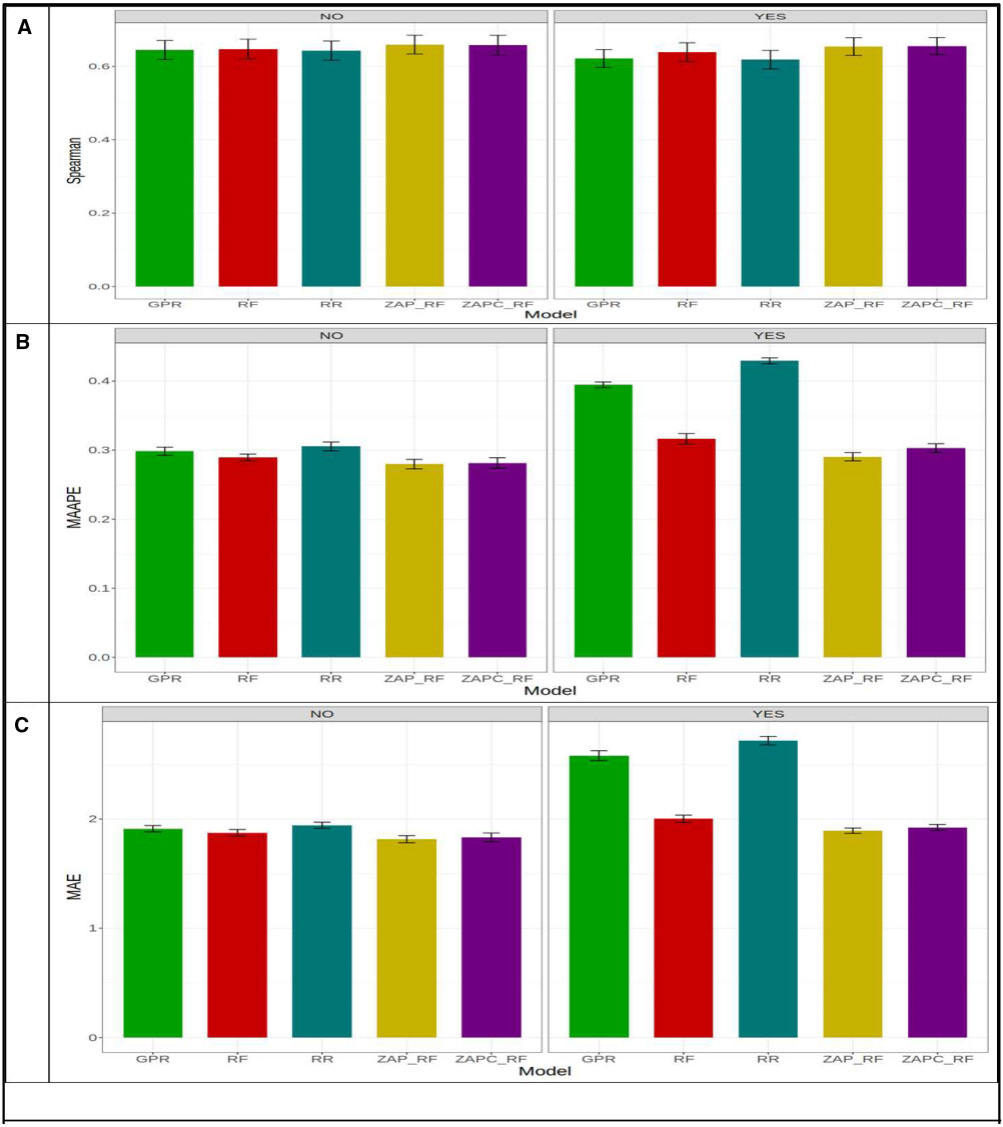
Prediction performance in terms of average Spearman’s correlation (Spearman; A), mean arctangent absolute percentage error (MAAPE; B) and mean absolute error of prediction (MAE; C) of the five models (GPR, RF, RR, ZAP_RF, ZAPC_RF) across environments in **dataset 2** for trait SN. NO in the plots means that the genotype×environment (GE) interaction was ignored, while YES means that the GE interaction term was taken into account.

Across-environments, taking into account the GE interaction, the best model in terms of Spearman’s correlation was ZAPC_RF (Spearman = 0.655) and the worst was model RR (Spearman = 0.618), and the ZAPC_RF outperformed the RR model by $\left(0.655\;-\;0.618\right)\times \frac{100}{0.655}=5.61\%$ (Figure 12A). In terms of MAAPE, the best and worst models were also the ZAP_RF (MAAPE = 0.290) and the RR (MAAPE = 0.429) models, respectively, and the best outperformed the worst by $\left(0.429\;-\;0.290\right)\times \frac{100}{0.290}=47.84\%$ (Figure 12B), while in terms of MAE, the ZAP_RF (best, with MAE = 1.892) outperformed the RR (worst, with MAE = 2.717) model by $\left(2.717\;-\;1.892\right)\times \frac{100}{1.892}=43.54\%$ (Figure 12C). While ignoring the GE term, in terms of Spearman’s correlation the best model was also the ZAP_RF (Spearman = 0.659) and the worst was also RR (Spearman = 0.643) and the ZAP_RF was superior to the RR model by $\left(0.659\;-\;0.643\right)\times \frac{100}{0.659}=2.51\%$ (Figure 12A). In terms of MAAPE (Figure 12B) and MAE (Figure 12C), the ZAP_RF (MAAPE = 0.279, MAE = 1.815) was also the best model and the RR (MAAPE = 0.305, MAE = 1.943) was the worst, and the ZAP_RF outperformed the RR by $\left(0.305\;-\;0.279\right)\times \frac{100}{0.279}=9.15\%$ in terms of MAAPE and by $\left(1.943\;-\;1.815\right)\times \frac{100}{1.815}=7.02\%$ in terms of MAE.

**Figure 13:**
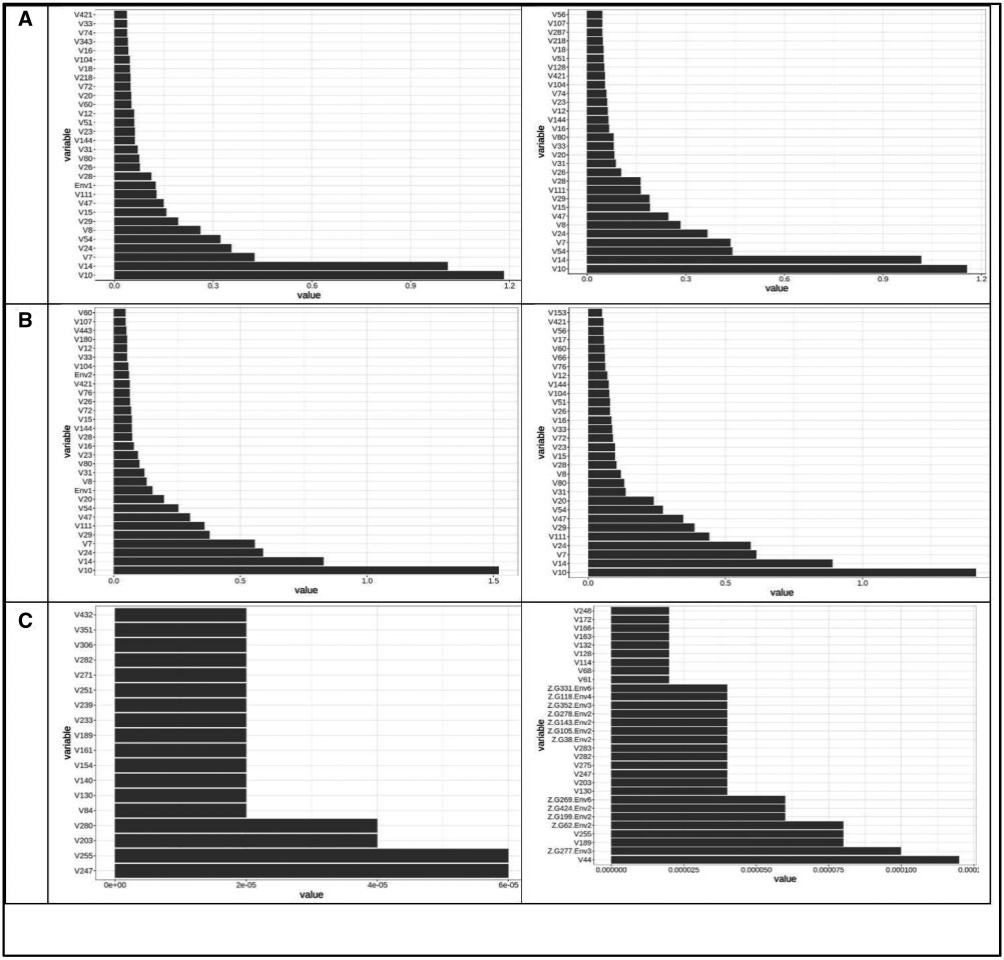
Predictor importance for trait SN in **dataset 2** under conventional random forest (A) and under the zero altered Poisson random forest for trait SN (B and C). The first column contains the results without interaction (NO) and the second column contains the results with interaction (YES).

The 30 most important VIM for the conventional random forest model (Figure 13A) and for the ZAP_RF model (Figure 13B, truncated part; Figure 13C, zero part) are given for trait SN in **dataset** 2. The three most important predictors for the conventional RF model were: V10, V14 and V7 (without GE interaction) and V10, V14 and V54 (with GE interaction). For the ZAP_RF model under the truncated part, the most important predictors were: V10, V14 and V24 (without the GE term) and V10, V14 and V7 (with the GE interaction term). Under the zero part, V247, V55 and V203 were the most important predictors ignoring the GE interaction, while with the GE interaction, the most important predictors were V44, Z.G277.Env3, and V189.

## Discussion

Due to the fact that there is no universal model that works in all circumstances, many statistical machine learning models have been adopted for genomic prediction. Random forest is one of the models adopted for genomic prediction with many successful applications (Sarkar *et al.* 2015; Stephan *et al.* 2015; Naderi, *et al.* 2016; Waldmann 2016; Li *et al.* 2018).

Some of the reasons for the increased popularity of random forests are: (1) they require very simple input preparation and can handle binary, categorical and numerical independent variables without the need for any preprocessing like scaling, (2) they perform implicit variable selection and provide a ranking of predictor (feature) importance, (3) they are inexpensive in terms of computational resources needed for its training since there are few hyper-parameters that need to be tuned (number of trees, number of features sampled and number of samples in the final nodes) and due to the fact that instead of working directly with all independent variables simultaneously each time, they use only a fraction of the independent variables, (4) some algorithms can beat random forests, but it is never by much, and other algorithms many times take much longer to build and tune than an RF model, (5) contrary to deep neural networks that are really hard to build, it is really hard to build a bad random forest, since it depends on very few hyper-parameters and some of them are not very sensitive, which means that a lot of tweaking and fiddling is not required to get a decent random forest model, (6) RFs are very versatile since they can deal with continuous, binary and categorical response variables, (7) they have a very simple learning algorithm, (8) they are easy to implement since there are many free and open-source implementations, and (9) RF parallelization is possible because each tree is grown independently.

The model originally proposed for estimation purposes by Mathlouthi *et al.* (2019) expanded the versatility of the random forest algorithm since ZAP_RF and ZAPC_RF are appropriate for count data with excess zeros. The main advantage of these methods is their flexibility, meaning they can adapt to the data at hand without having to specify a parametric form. We found that the proposed methods outperformed Ridge regression and Poisson Ridge regression and slightly outperformed the conventional random forest. For this reason, the proposed methods contribute to the lack of efficient algorithms for dealing with count data with excess zeros. The previously mentioned advantages of conventional RF are inherited by the proposed methods since the only difference between the conventional RF and the proposed zero altered Poisson random forest models is that instead of training only a conventional random forest model with the sum of squared errors (least squares) as splitting criteria, now two random forest models are trained, one for the excess of zeros (with conventional splitting criteria for binary outcomes like the Gini index or log-likelihood of Bernoulli distribution) and another for counts larger than zero that use the log-likelihood of zero truncated Poisson distribution as splitting criteria. This change in using two models instead of one allows the conventional random forest to be modified to deal better with count data with excess zeros. Also, the proposed zero altered Poisson random forest methods allow reporting the important features (predictors), but instead of one graph, two are generated, one for the zero-altered part that shows which features are the most important to the counts with excess zeros and the other for the remaining counts (1, 2,…). These two graphs of important predictors are very useful to gain insight into the biological meaning of the most important predictors.

The proposed zero altered Poisson random forest methods (ZAP_RF and ZAPC_RF) belong to the category of ensemble regression tree models, that by their nature it is difficult to evaluate the effect of each predictor. This means that these methods differ from parametric models (*e.g.*, a linear mixed model) for GWAS since they do not provide the parameter estimates and p-values (for significance) for measuring the degree of importance of each predictor. However, many other non-parametric models allow calculating variable importance values (denoted as VIM) to indicate the contributions of individual predictors to the prediction error. Figures 4, 7, 10, and 13 show the distribution profiles of the VIM values of the ranked predictors (from the most important to the least important ones) for RF analyses and for the proposed methods. The larger the predictor VIM value, the more important a predictor is. Most of the predictors were found to have either very small positive influence or no effect on the VIM values in RF and the proposed methods. Also, since the proposed zero altered Poisson random forest models (ZAP_RF and ZAPC_RF) were built with two models (a truncated and zero part), they provide two plots for the VIM values, one for the truncated part and another for the zero part, indicating that different predictors influence each model. These plots are of paramount importance because they allow identifying which predictors play the most important role in the prediction of the response variable of interest.

It is important to point out that under a univariate Poisson regression, the *inverse link function* is equal to $\mu_{i}=\exp(\eta +\sum_{j=1}^{p}x_{ij}\beta_{j})$. However, if we change the inverse link function to $\mu_{i}=\eta +\sum_{j=1}^{p}x_{ij}\beta_{j},\;$that is, an identity inverse link function, and if we assume that $y_{i}∼\mathrm{Normal}\left(\mu_{i}=\eta +\sum_{j=1}^{p}x_{ij}\beta_{j},\sigma^{2}\;\right)$, we move from a univariate Poisson regression to a univariate Gaussian regression model. However, there is a lot of empirical evidence that for count response variables, the Poisson regression model should be preferred since it guarantees that all predictions are non-negative (which is not guaranteed with a normal model) (Montesinos-López *et al.* 2015, 2016, 2017). When the Gaussian regression is used instead of Poisson regression, negative outputs of the Gaussian regression must be truncated to zero, and it is unclear how this affects the optimality of the predictive distribution (Montesinos-López *et al.* 2015, 2016, 2017). However, in terms of prediction performance there is also evidence that many times (for particular datasets) using a Gaussian model gives similar prediction performance to a Poisson regression; however, when the count contains an excess of zeros, the normal approximation fails to capture the excess of zeros, and for this reason, improved versions of Poisson regression such as the zero-inflated Poisson and zero altered Poisson regression, are used. For this reason, the goal of our proposed method is to improve the prediction performance of counts in the presence of an excess of zeros.

Also, as conventional RF, the individual decision trees generated by the proposed methods (ZAP_RF and ZAPC_RF) are prone to overfitting (that is, they have high variance and low bias), but by resampling the data many times to create a large number of un-pruned decision trees, the accuracy of prediction based on sample data is improved due to the fact that the variance component is reduced. Also, the proposed methods do not differentiate between random (lines) and fixed effects (environments) since they are non-parametric models; for this reason, the environmental, genotypic and genotypic ×environmental effects used in the inputs are treated as additional predictors in the model, that also influence the response variable, as shown in the plots of predictor importance for each trait (Figures 4, 7, 10, and 13).

## Conclusion

In this paper, a zero altered Poisson random forest model was evaluated for genomic prediction. This model is a modified random forest that instead of fitting only one RF model, two random forest models are implemented: one for the zero counts (with splitting criteria using the Gini index) and another for the counts larger than zero (with a splitting criterion based on the log-likelihood of a zero truncated Poisson distribution). The two versions of the proposed model for excess zeros (ZAP_RF and ZAPC_RF) were compared in terms of prediction performance with Ridge regression for continuous outcomes, Poisson Ridge regression and conventional random forest. Our results suggest that the two versions of the proposed zero altered Poisson random forest model most of the time was the best in terms of prediction performance and clearly outperformed Ridge regression and Poisson Ridge regression, but produced only a slight improvement over the conventional random forest model. However, we observed that in **dataset 1**, which contains a larger percentage of excess zeros, the proposed model was clearly better than all models. For this reason, we also provide the cv.zap.rf() function to implement in R the proposed models to enable other scientists with other real data to benchmark the prediction performance of the proposed methods. Finally, we encourage the use of the proposed zero altered random forest models because their implementation is straightforward using the proposed cv.zap.rf() function in the R statistical software, and they produce very competitive predictions like the conventional random forest model.

## Appendix A: cv.zap.rf () function to implement in R the zero altered Poisson random forest.

rm(list = ls(all = TRUE))

devtools::install_github(“brandon-mosqueda/randomForestSRC”)

library(randomForestSRC)

library(dplyr)

load(“Data_Real_Count.RData,” verbose = TRUE)

Pheno <- rename(Pheno, Line = “GID,” Env = “Loc,” Response = “y”)

Geno <- as.data.frame(G)

Geno$Line <- unique(Pheno$Line)

Results <- cv.zap.rf(

 Pheno, Geno = Geno, Markers = NULL, with_interaction = TRUE, mult_env_anal = TRUE,

 ntree_theta = c(100, 300, 500), mtry_theta = c(0.15, 0.30, 0.45),

 nodesize_theta = c(2, 5, 15), ntree_lambda = c(100, 300, 500),

 mtry_lambda = c(0.15, 0.30, 0.45), nodesize_lambda = c(2, 5, 15),

 importance = TRUE, type = c(“original”), loss_function = mse,

 cross_validation = “k_fold,” number_of_folds = 5, proportion_of_testing = 0.2, type_of_tuning = “local,” tuning_cross_validation = “k_fold,” tuning_number_of_folds = 5, tuning_proportion_of_testing = 0.2, sample_proportion = 1, digits = 4, seed = NULL, results_dir = “zap_random_forest_results,” verbose = TRUE)

 # View individual predictions per fold

 Results$All

This function, cv.zap.rf(), allows as input a genomic relationship matrix (GRM) or marker information directly. Also, by default the input takes into account the information of environments, genotypes and genotype by environment interaction (with_interaction = TRUE), but if you specify FALSE in with_interaction =FALSE, it only takes into account in the predictor the environment and genotypes. Also, if you want to perform single environment analysis using only genotypic information in the predictor, you need to specify mult_env_anal =FALSE. With type = “original,” the ZAP_RF is implemented, but with type = “custom” the ZAPC_RF is implemented. Two types of cross-validation are available: k_fold and random_partition; in the random_partition you need to specify the number of partitions in number_of_folds and the proportion for testing in each partition in proportion_of_testing. For the tuning process, you also need to specify the type of cross-validation and also k_fold and the type random_partition; sample_proportion = 1 use all the information in the grid of hyper-parameters (a value between 0 and 1 is possible). For example, when sample_proportion = 0.2 is used, only 20% of all combinations available in the grid will be evaluated to choose the best hyper-parameters. Finally, in results_dir =, you need to write the name of the directory where the outcomes should be saved.
